# A Novel Approach for Characterizing Microsatellite Instability in Cancer Cells

**DOI:** 10.1371/journal.pone.0063056

**Published:** 2013-05-06

**Authors:** Yuheng Lu, T. David Soong, Olivier Elemento

**Affiliations:** 1 Department of Physiology and Biophysics, Weill Cornell Medical College, Cornell University, New York, New York, United States of America; 2 Institute of Computational Biomedicine, Weill Cornell Medical College, Cornell University, New York, New York, United States of America; Albert Einstein College of Medicne, United States of America

## Abstract

Microsatellite instability (MSI) is characterized by the expansion or contraction of DNA repeat tracts as a consequence of DNA mismatch repair deficiency (MMRD). Accurate detection of MSI in cancer cells is important since MSI is associated with several cancer subtypes and can help inform therapeutic decisions. Although experimental assays have been developed to detect MSI, they typically depend on a small number of known microsatellite loci or mismatch repair genes and have limited reliability. Here, we report a novel genome-wide approach for MSI detection based on the global detection of insertions and deletions (indels) in microsatellites found in expressed genes. Our large-scale analyses of 20 cancer cell lines and 123 normal individuals revealed striking indel features associated with MSI: there is a significant increase of short microsatellite deletions in MSI samples compared to microsatellite stable (MSS) ones, suggesting a mechanistic bias of repair efficiency between insertions and deletions in normal human cells. By incorporating this observation into our MSI scoring metric, we show that our approach can correctly distinguish between MSI and MSS cancer cell lines. Moreover, when we applied this approach to primal tumor samples, our metric is also well consistent with diagnosed MSI status. Thus, our study offers new insight into DNA mismatch repair system, and also provides a novel MSI diagnosis method for clinical oncology with better reliability.

## Introduction

In normal cells, mismatch repair (MMR) system provides a highly efficient mechanism for correcting errors that occur during DNA replication. When impaired, e.g. through inactivation of human mismatch repair genes such as MLH1, MSH2 and MSH3, mismatch repair deficiency leads to uncorrected insertions/deletions (indels), particularly in microsatellites where a short sequence unit (one to six nucleotides long) is repeated multiple times [1].

Microsatellite instability (MSI) refers to the genetically aberrant condition in which microsatellite alleles in genome gain or lose repeat units at much higher frequency than in normal cells. The normal condition is often referred to as microsatellite stable, or MSS. Widespread MSI usually indicates mismatch repair deficiency (MMRD), which can cause accumulation of mutations in cancer-related genes and lead to carcinogenesis and tumor progression. Accordingly, MSI is frequently observed in several types of cancers, most notably in colon cancer [2] and prostate cancer [3]. The presence of MSI can be used as a marker for specific tumor subtypes and can predict sensitivity to chemotherapy [1]. Moreover, MSI generates significant genetic heterogeneity and can be used for other purposes such as the isolation of drug resistant clones and the subsequent characterization of drug resistance mechanisms [4].

Currently, several assays exist for the detection of MSI, including those looking for mutations in MMR genes, measuring their expression, or looking for unit number alterations in a set of microsatellites frequently affected by MSI [5], [6]. However, these assays are not always reliable. For example, two studies using different assays provided opposite results about the MSI status of the PC3 prostate cancer cell line [3], [7]. Moreover, commonly used MSI markers are cell type specific. For instance, it has been reported that commonly used markers in colon cancer have low sensitivity in acute myeloid leukemia [8].

Many other factors can negatively affect the accuracy of available MSI detection techniques. Methods that use loss of expression or mutation of known MMR genes may be hampered by the complexity of the MMR system, which consists of multiple genes and possibly other uncharacterized ones. The low sensitivity of unit number based approaches may be explained by the random nature of MSI: MMRD may affect different sets of microsatellites in different individuals. That may explain why the limited sets of markers used in current MSI detection assays sometimes give false negatives.

To overcome such limitations, we developed a novel method that improves the sensitivity of MSI detection by incorporating all detectable microsatellites across the genome characterized by next-generation sequencing. We chose to base our analysis on RNA-Seq, as it is a relatively mature next-generation sequencing technique and has already been widely performed for various applications. Although short-read sequences pose challenges for mapping and characterizing microsatellites, we have overcome these issues and demonstrated the reliability of our genome-wide scanning approach for MSI detection. In addition to basing detection on a vastly increased number of microsatellite indels in MSI cells, our approach exploits a phenomenon so far only reported in yeast but that we observed in human cells in this study: indel length distributions in MSI and MSS samples are significantly different [9]. Genome-wide detection of microsatellite indels avoids shortcomings of limited marker set and provides higher sensitivity for MSI detection. Besides being sensitive, our approach does not require a matched germline control from the same individual, and can therefore be applied to cancer cell lines.

## Materials and Methods

### Datasets

In this study, we used RNA-seq datasets for 20 different cancer cell lines in order to investigate MSI frequencies in different cancer types. All cancer cell line datasets were obtained from published studies, and the MSI status of many of these cell lines has already been reported (although for some cell lines results from different studies conflict). Table 1 shows the MSI status information and sources of RNA-seq datasets. We also used two published RNA-seq studies of HapMap lymphoblastoid samples collected from 69 Nigerian [10] and 54 European individuals [11] as controls to define MSI status in normal human cells, as MSI are not expected in those samples. We also analyzed paired colon cancer tumor and normal RNA-Seq samples from 14 patients diagnosed with MSI tumors and 14 patients diagnosed with MSS tumors [12].

**Table 1:** Cancer cell lines used in this study and their MSI status described in previous studies.

Cell line	Tissue type	MSI status	Source of RNA-seq data
HCT116	Colon	+ [24]	[4]
MIP101	Colon	+ [25]	[26]
DU145	Prostate	+ [24]	[27]
LNCaP	Prostate	+ [3], [7]	[28]
22Rv1	Prostate	+ [3], [7]	[28]
MDA-PCa-2b	Prostate	+ [3], [7]	[28]
CWR22	Prostate	+ [3], [7]	[28]
VCaP	Prostate	?	[28]
PC3	Prostate	* [3], [7]	[28]
NCI-H660	Prostate	−[3], [7]	[28]
MCF-7	Breast	−[21]	[29]
T47D	Breast	−[21]	[30]
KPL-4	Breast	?	[29]
SK-BR-3	Breast	?	[29]
BT-474	Breast	?	[29]
501 Mel	Melanoma	?	[31]
K562	Leukemia	−[32]	[31]
Saos-2	Osteosarcoma	?	[33]
MKN-28	Gastric	?	[34]
MKN-45	Gastric	−[24]	[34]

+: MSI; −: MSS; ?: no direct evidence; *: results from different studies conflict.

### Detecting Microsatellite Indels

**Figure 1:**
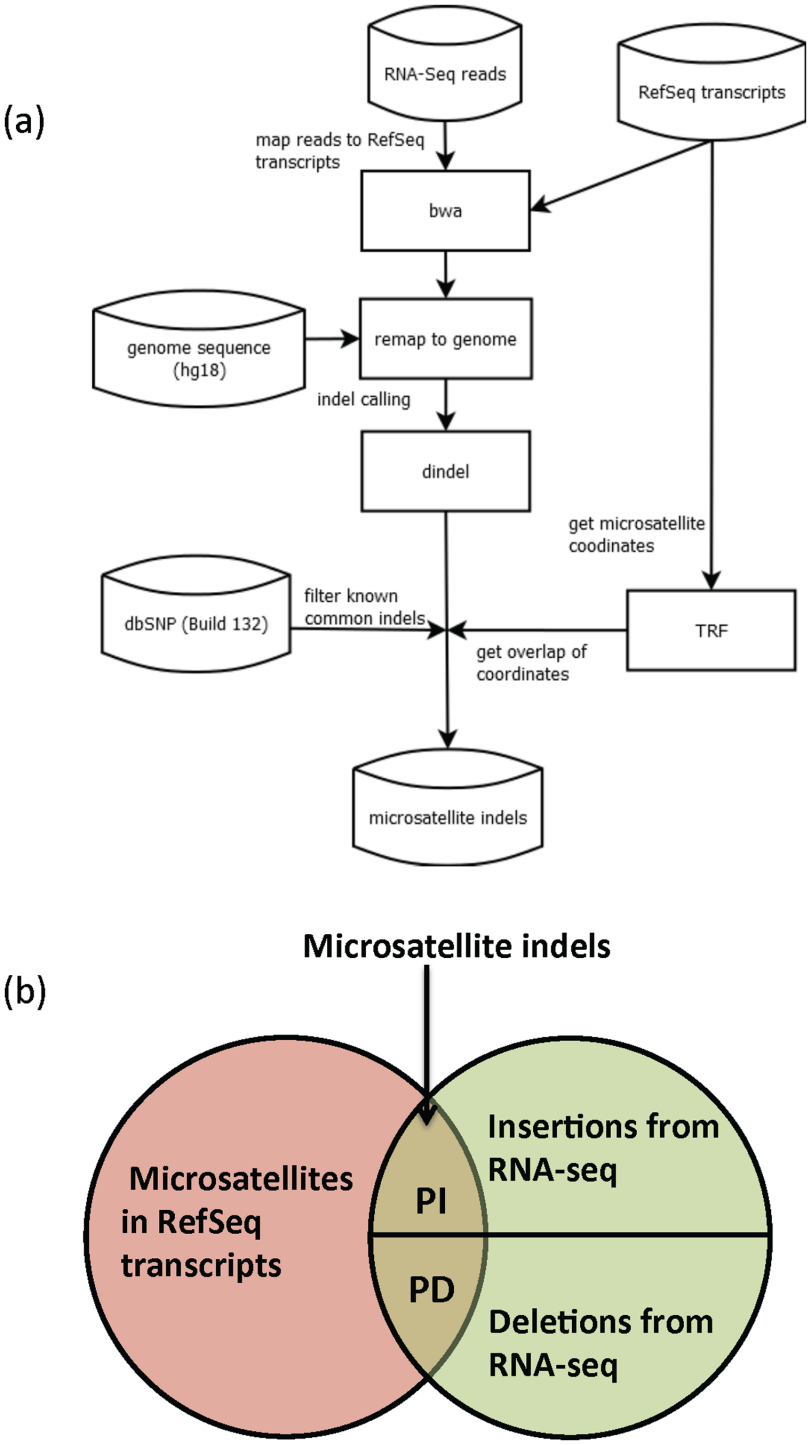
An outline of the study (a) MSI characterization pipeline used in this study (b) Definition of PI and PD. PI refers to the proportion of insertions in microsatellites over all insertions, and PD refers to the proportion of deletions in microsatellites over all deletions.

We first extracted microsatellites in all human RefSeq transcripts using Tandem Repeats Finder (TRF) [13]. In this study microsatellites were defined as tandem repeats with repeat units of 1 to 6 base pairs. For each RNA-seq dataset, we then aligned the short reads to RefSeq transcripts using BWA [14], which allows gapped-alignment. We used a maximum gap size of 20 bp for all analyses reported here. We also tested larger gap sizes but they did not increase the number of detected indels in the analyses that follow. For the rest of parameters we used BWA default parameters. We then used DINDEL [15] to call indels from aligned reads. From the output of dindel, we filter out common indels listed in Single Nucleotide Polymorphism database (dbSNP; Build 132) [16]. Finally, we compared the coordinates of indels to the coordinates of microsatellites found by TRF and identified indels within microsatellites (**Figure 1a**).

### Quantifying MSI Using the MSI-seq Index

We evaluated several MSI measures based on the number of indels and microsatellites determined by our analysis pipeline. These measures included the proportion of microsatellite insertions over all insertions (denoted as PI), and the proportion of microsatellite deletions over all deletions (denoted as PD; **Figure 1b**). We also evaluated PI/PD, since our results, in agreement with a previous study in yeast [9], suggested that MMRD might alter the relative rates of insertions and deletions. PI/PD is also referred as MSI-seq index in this study.

### Expression Profiling of MMR Related Genes

To compare our approach with methods using the expression level and mutation status of MMR system components, we determined the expression level of MMR genes (including MLH1, MLH3, MSH2, MSH3, MSH4, MSH5, MSH6, PMS1 and PMS2) in cancer cell lines from the RNA-seq data. We used Cufflinks [17] to compute normalized expression levels measured in fragments per kilobase of transcript per million reads (FPKM). We also looked for indels in MMR genes using the DINDEL results (not restricted to microsatellites). Finally, we looked for single nucleotide variants in the same MMR genes using SNVseeqer [4], [18], [19]. As we did for indel calling, we only retained SNVs not listed in dbSNP for further analysis.

## Results

### Indel Detection in RNA-seq Data of MSI/MSS Samples

**Figure 2:**
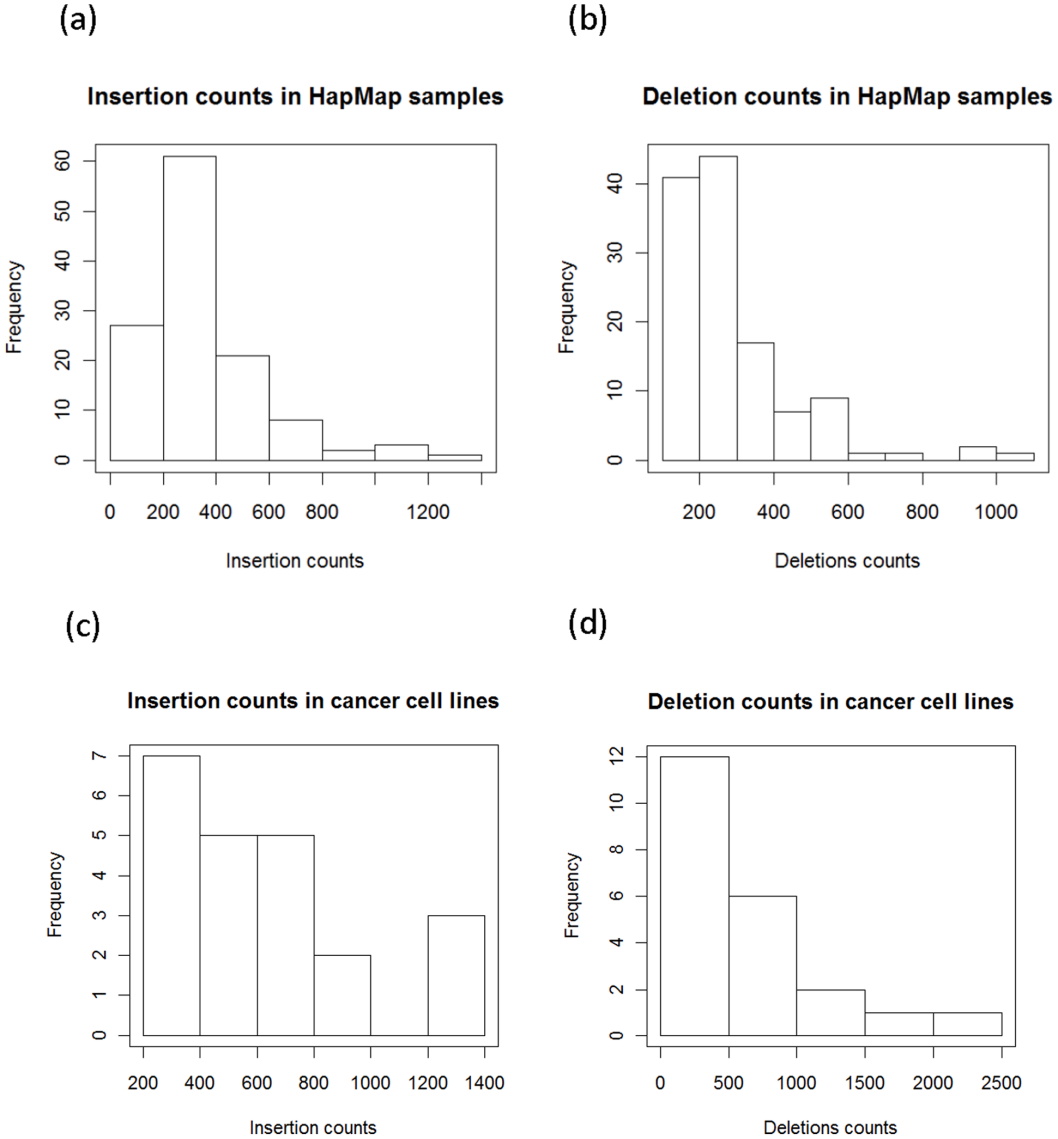
Indel counts from RNA-Seq.Y axis shows the number of samples. (a) HapMap samples (b) cancer cell lines.

We first sought to identify indels within the RNA-seq samples used in this study (MSI, MSS, HapMap). After short-read alignment using BWA, we used DINDEL to call indels in our 20 cancer cell lines and 123 HapMap RNA-seq datasets. From the DINDEL outputs, we filtered out indels listed in Single Nucleotide Polymorphism database (dbSNP; Build 132) [16]. After filtering, absolute indel counts vary from around 200 to more than 2000 (**Figure 2**).

### Different Distributions of Microsatellite Indel Alterations in MSI/MSS Samples

**Figure 3:**
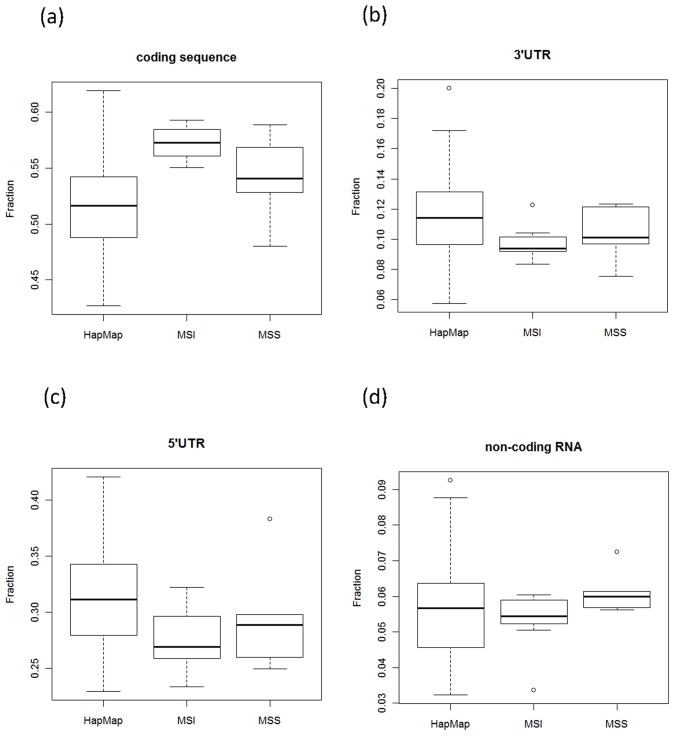
Proportions of microsatellite indels in different regions of transcripts. (a) Coding sequences (b) 3′UTR (c) 5′UTR (d) Non-coding RNA.

Next, we sought to determine the frequency with which microsatellite sequences are altered by indels in each RNA-seq sample. A total of 505,657 microsatellites were found in 32,199 Refseq transcript sequences using TRF. We then determined the number of microsatellites altered by at least one indel in each RNA-seq sample. This quantity ranges from 54 to 482 in HapMap samples, and from 77 to 1454 in cancer cell lines. In all following analyses, we only study indels within microsatellites. In each sample, we then determined the proportion of microsatellites altered by indels located in 5′ UTR, coding sequence, 3′ UTR or non-coding RNAs, and determined whether these proportions are significantly different between MSI and HapMap samples and between MSS and HapMap samples. We observed a significant increase in the proportion of indels in MSI samples’ coding sequences (p = 1e–5, Student’s t-test; **Figure 3a**) when compared to HapMap samples, while the proportion remained similar in MSS samples (p = 0.15, Student’s t-test). Corresponding to this increase, the proportion of microsatellite indels in other regions was lower in MSI samples compared to HapMap (5′ UTR: p = 0.0149; 3′ UTR: p = 0.0108) while there is no difference between MSS samples and HapMap (p>0.05; **Figure 3b–c**). In non-coding RNAs, we observed no significant differences between HapMap, MSI and MSS (p>0.05; **Figure 3d**). The prevalence of microsatellite indels in coding regions of MSI (and to some extent in MSS) cancer cells suggest that these indels might lead to a selective advantage to cancer cells, consistent with findings in other organisms [20].

**Figure 4:**
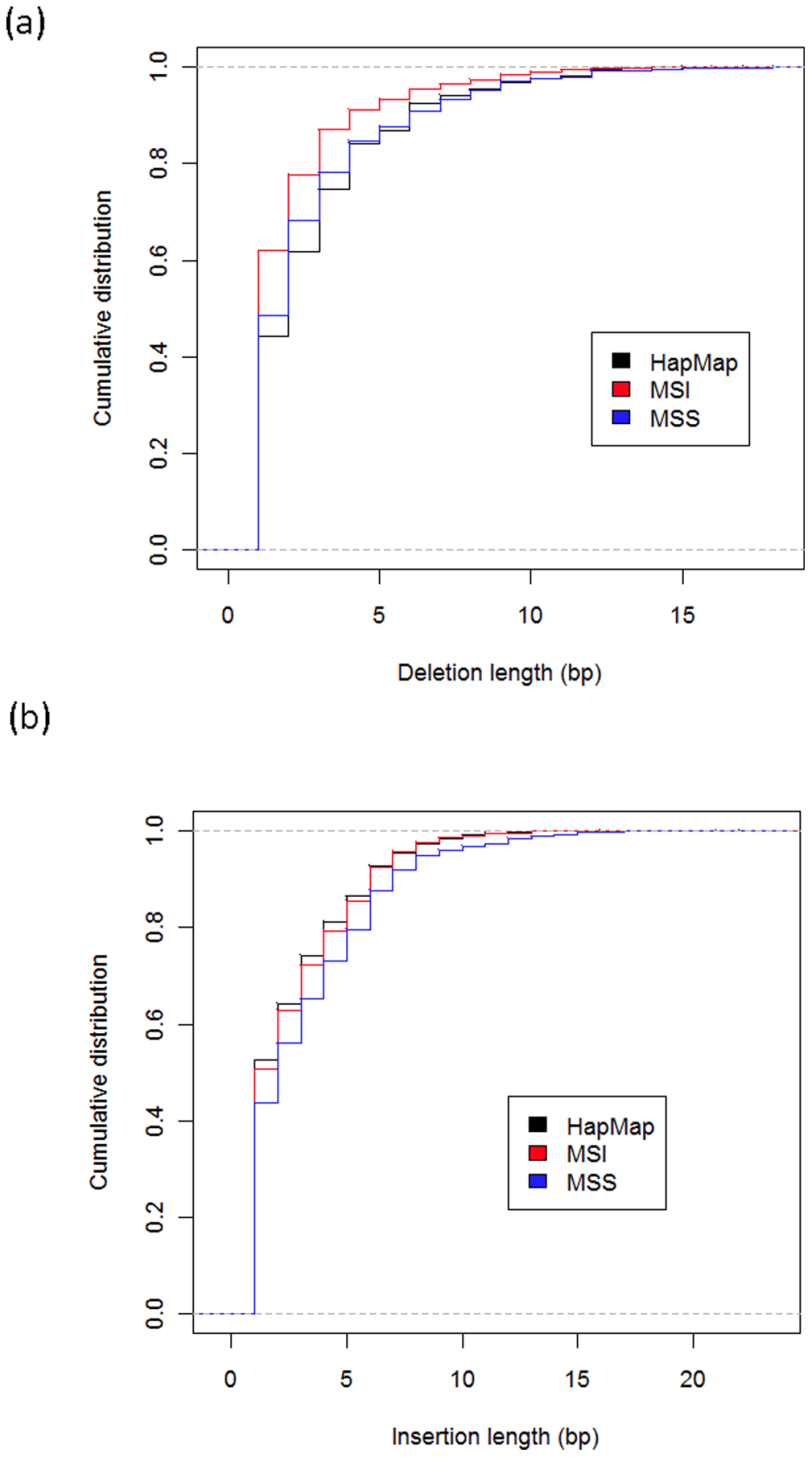
Cumulative distribution of microsatellite indel lengths for cancer cell lines and HapMap samples. (a) Microsatellite deletions (b) Microsatellite insertions.

In a previous study in yeast, it was found that after mutating DNA mismatch repair proteins, there was a significant increase in the number of short deletions in microsatellites, while the number of insertions in microsatellites did not change significantly [9]. Inspired by those results, we also studied the distribution of detected microsatellite indel lengths in MSI/MSS samples. We noticed that short deletions of 1 bp are more frequent MSI cell lines than they are in HapMap samples (**Figure 4a**). To further investigate this observation, we compared the distributions of insertion and deletions obtained from each of the 7 MSI cancer cell lines and 5 MSS cell lines to the overall distributions of insertion and deletions from all 123 HapMap controls. Kolmogorov–Smirnov test showed that length distributions of microsatellites deletions in MSI samples were significantly different from HapMap samples (p<0.05). In contrast, all except one MSS samples show no significant difference in deletion lengths (p>0.05). The distributions of insertion lengths, on the other hand, were not significantly different between the 3 groups (**Figure 4b**). These observations suggest that deficiency with the mismatch repair machinery that is associated with microsatellite instability preferentially give rise to short deletions.

### The MSI-seq Index Correctly Predicts MMRD Cell Lines

**Figure 5:**
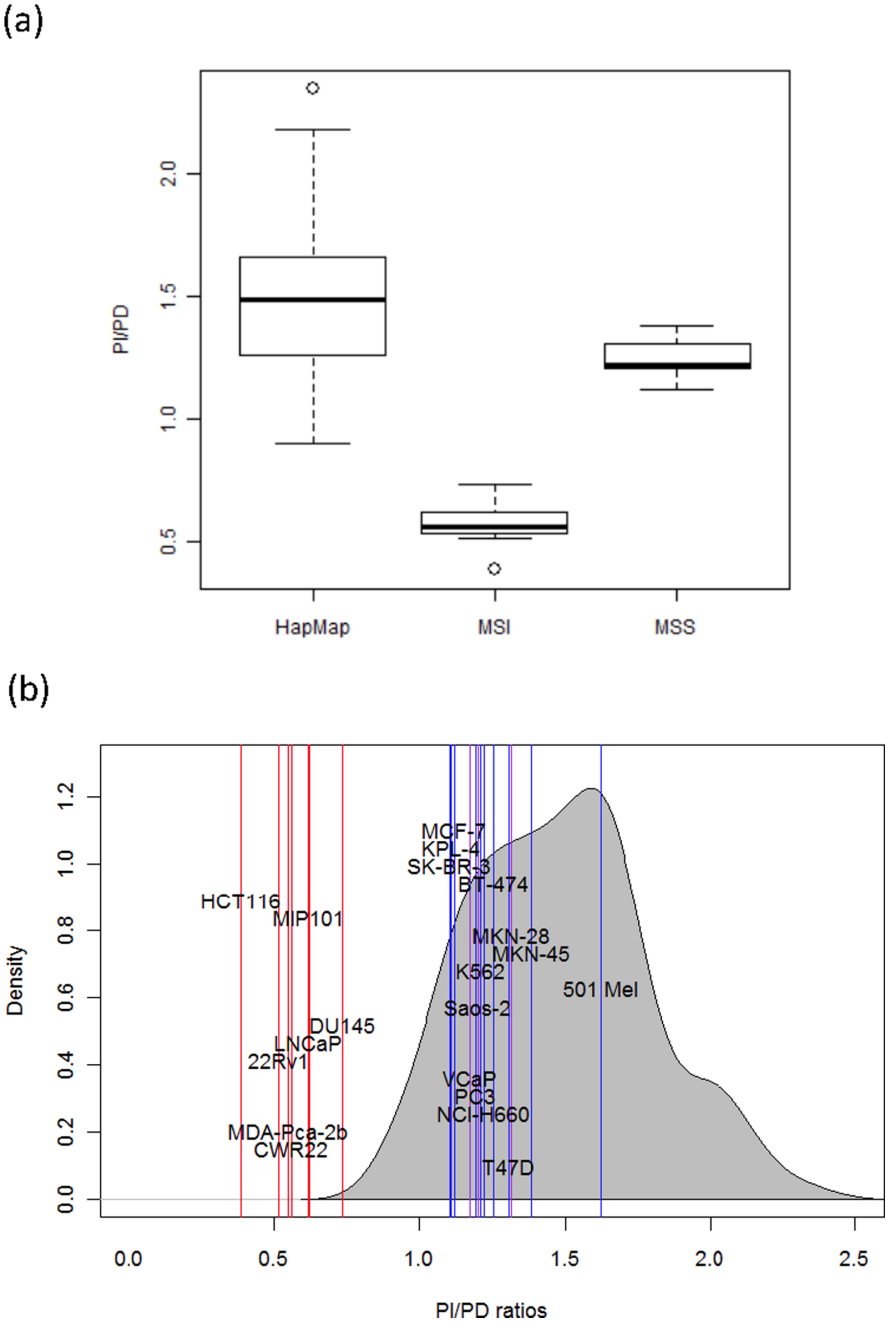
MSI-seq index correctly reflects the MSI statuses of samples. (a) Comparison between the PI/PD ratios of HapMap samples, MSI cancer cell lines and MSS cancer cell lines (b) PI/PD ratios for all HapMap samples and cancer cell lines. HapMap samples’ PI/PD ratios are represented by the empirical distribution density curve. The ratios for known cell lines are plotted in red vertical lines. Known MSS cell lines are plotted in blue, and all the other cell lines are plotted in purple.

Our original goal was to identify a genome-wide index or measure that reliably distinguishes MSI samples from MSS samples. We initially investigated two possible measures: the proportion of microsatellite insertions over all insertions (denoted as PI), and the proportion of microsatellite deletions over all deletions (denoted as PD; **Figure 1b**). The analysis in previous section revealed that compared to normal human cells, number of short deletions significantly increased in MSI cell lines but not in microsatellite stable (MSS) cancer cell lines. Due to this difference, PD discriminated between MSI and MSS samples, albeit not perfectly (data not shown). In contrast, although PI is unable to discriminate between the two types of cells, it still reflects the absolute number of microsatellite indels. As a result, normalizing PD with PI can make MSI statuses of different samples more comparable. We thus investigated the ratio between two proportions as an alternative index to discriminate between MSI and MSS cell lines. When we calculated PI/PD for MSI and MSS cancer cell lines, we observed significant differences between the two groups (p = 2.4e–5, t-test), with MSI showing lower PI/PD (**Figure 5a**). As expected, PI/PD values were also significantly different between MSI and HapMap (p = 1.5e–10, t-test). The fact that MSI and MSS samples have statistically different PI/PD values does not mean that PI/PD is highly reliable at discriminating MSI and MSS. However, we further observed that PI/PD clearly separates MSI and MSS cell lines into two distinct groups (**Figure 5a**). Indeed, all cell lines that have been identified as MSI (Table 1) exhibit PI/PD ratios lower than 1. In contrast, MSS cell lines all have ratios larger than 1, similar to HapMap samples (**Figure 5a**). Those results demonstrate that the PI/PD ratio, which we refer to as MSI-seq index, can accurately predict MSI status in cancer cell lines, even spanning multiple cancer types.

The results above show that the MSI-seq index can reliably predict MSI status. We therefore applied our analysis to cell lines whose MSI status had not been characterized. In our study, all such cell lines fell in the range of HapMap samples (**Figure 5b**). Therefore, by our criteria, they are likely to be MSS cell lines. Predictions for some cell lines were supported by additional evidence. For instance, all three MSI-uncharacterized breast cancer cell lines are categorized as MSS. This result is consistent with previous studies suggesting that MSI may only play a minor role in the oncogenesis of breast tumors compared to other tumor types [21].

### Expression or Mutation of Known MMR Genes Fails to Reliably Predict MSI Status

We then sought to determine whether other simpler approaches based on expression or mutation of known MMR genes could predict MSI status. Previous findings have indeed suggested that inactivation of MMR genes could cause MMRD [1]. Therefore, in principle the expression profile of MMR-related genes could also predict MMRD and MSI. We looked at 7 MSI cell lines and 5 MSS cell lines with MSI statuses validated by previous studies.

**Figure 6:**
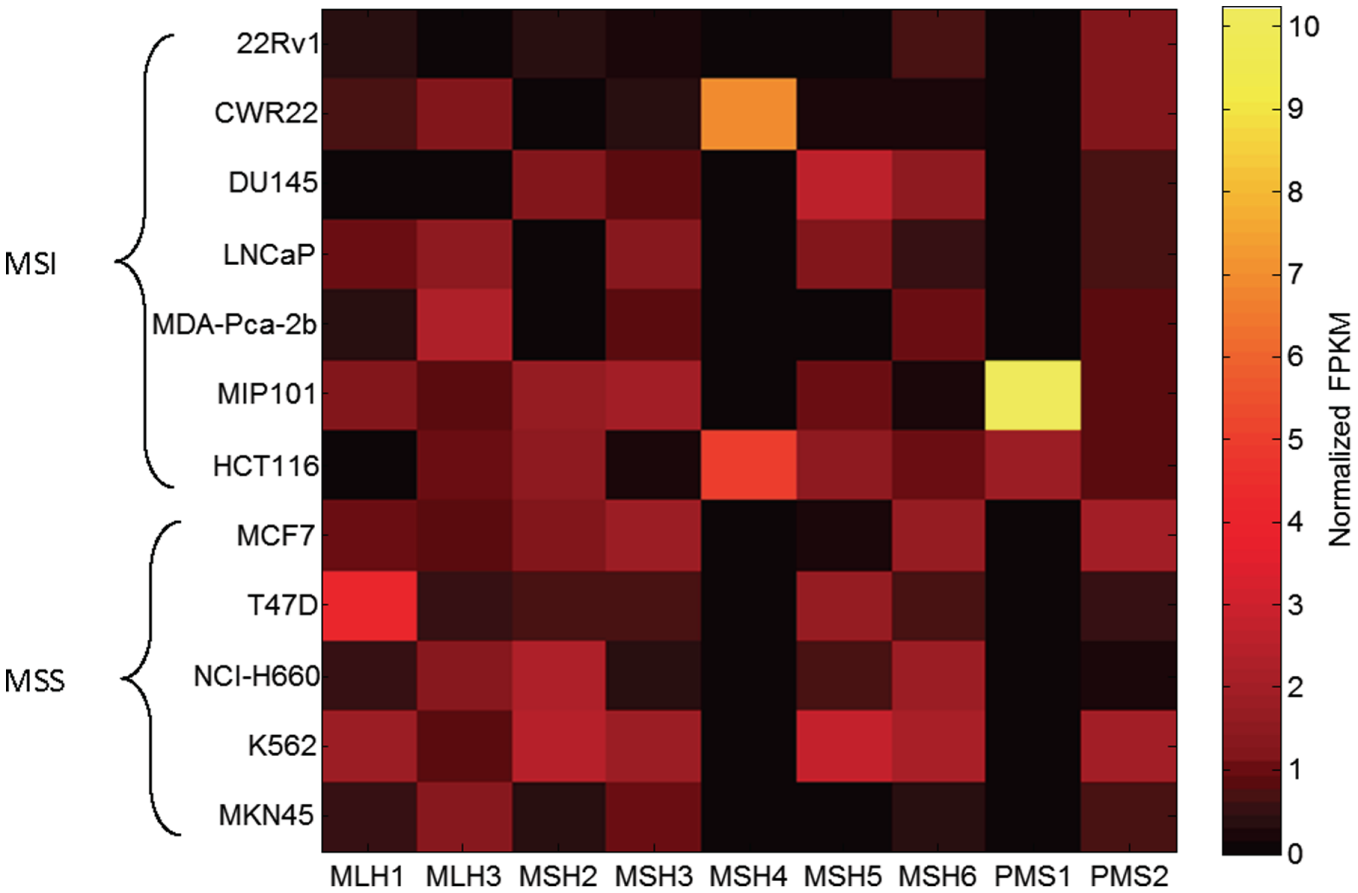
Expression levels of MMR genes shown in FPKM values for MSI/MSS cell lines. Values are normalized by the mean value of each column.

The expression levels of known MMR genes did not correlate with MSI status (**Figure 6**; FPKM values normalized by the mean value of each column). For instance, MLH1 expression is repressed in two cell lines, HCT116 and DU145. Such a loss is accompanied by the loss of MLH3 expression in DU145 and loss of MSH3 expression in HCT116, as previously reported [22]. In another MSI cell line LNCaP, however, all these genes are expressed in normal levels, while the repressed expression of MSH2 may be responsible for MSI [3], [23]. Analysis of point mutations and indels also showed lack of correlation with MSI status, with MSS samples showing missense variants in MMR genes while many MSI cells have no variants (**Table 2**). Therefore, unlike the MSI-seq index, neither MMR gene expression levels nor mutations are truly reliable for MSI detection.

**Table 2:** MMR gene alterations in cancer cell lines with determined MSI status.

	Status	MSH6	MSH2	MLH1	PMS1	MSH3	MSH4	MSH5	PMS2	MLH3
22Rv1	MSI	0	0	0	0	0	0	0	0	0
CWR22	MSI	**1**	0	1	0	**1**	0	0	1	0
DU145	MSI	2	1	0	0	**2**	0	0	1	0
LNCaP	MSI	1	0	0	0	3	0	0	1	1
MDA-Pca-2b	MSI	1	0	0	0	**3**	0	0	2	0
MIP101	MSI	1	0	3	0	3	0	1	2	0
HCT116	MSI	0	0	0	0	1	0	0	2	0
MCF7	MSS	0	1	0	0	2	0	0	1	1
T47D	MSS	0	0	0	0	0	0	0	0	0
NCI-H660	MSS	1	2	1	0	2	0	0	0	0
K562	MSS	**0**	0	1	0	1	0	3	1	0
MKN45	MSS	0	0	0	0	0	0	0	0	0

Counts indicate the number of missense mutations in MMR genes, and cells with text in bold correspond to indels found in MMR genes.

### Application to Clinical Samples

**Figure 7:**
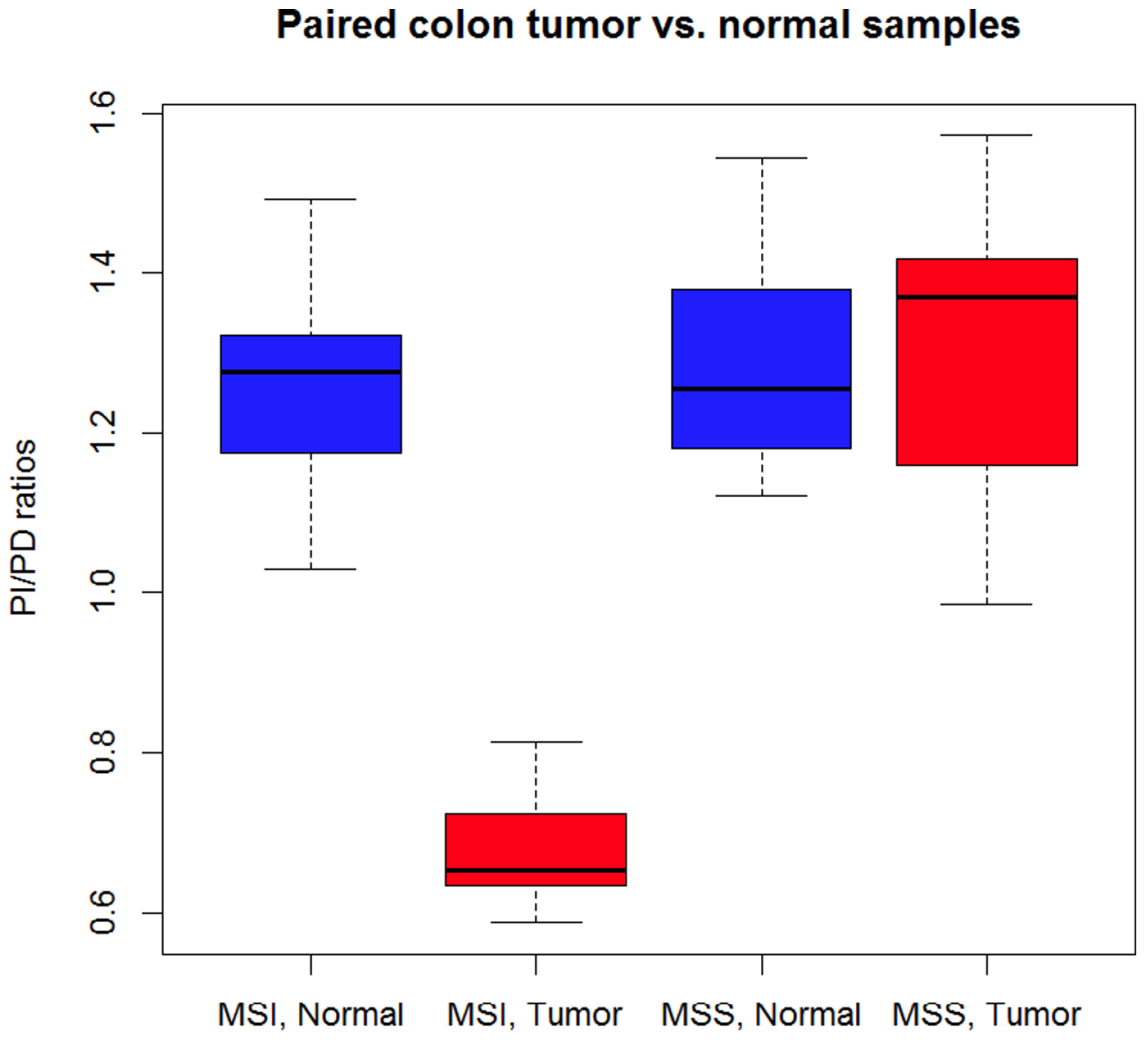
PI/PD ratios for paired tumor and normal samples from colon cancer patients. 14 are diagnosed as MSI and 14 are diagnosed as MSS.

We then tested whether our approach can predict MSI status in primary tumors. We analyzed paired colon cancer tumor and normal RNA-Seq samples from 14 patients diagnosed with MSI tumors and 14 patients diagnosed with MSS tumors [12]. The ratios for MSI tumor samples range from 0.630 to 0.814, while the ratios for MSS tumor samples range from 0.986 to 1.573. Therefore a threshold around 0.9 will be able to produce accurate MSI status predictions, which is similar to what we have observed in cancer cell lines. Moreover, the ratios for MSI tumor samples showed a significant difference compared to paired normal samples (p = 5.57e-11; t-test), while there is no difference between MSS tumor and normal samples (p = 0.6438; t-test) (**Figure 7**). This result indicates that our method not only works in cancer cell lines, but is also effective to detect MSI status in primary tumors.

## Discussion

In this study, we have introduced a novel and reliable index (MSI-seq) for detecting microsatellite instability using transcriptome sequencing data. Unlike other approaches that are limited to querying a small number of genes or microsatellite regions, our method integrates indel data from a large number of expressed microsatellites. Our approach does not depend on germline DNA and is therefore equally applicable to cancer cell lines and primary samples. We have shown that our approach is more accurate than approaches based on the detection of point mutation or expression changes in mismatch repair genes. Another advantage of our approach is that the MSI-seq index provides a continuous measure of MSI. In previous studies, MSI was often treated as an all-or-nothing event. Traditional assays could only tell whether a sample is MSI or MSS. However, in biologically relevant cases, the MSI status of samples are likely to span a continuous spectrum, and the extent of MSI may have therapeutic implications. Therefore, the MSI-seq index (PI/PD ratio) used in this study may be a promising candidate for quantifying the relative MSI degree of a sample.

During the course of our investigations regarding indels in cancer cell lines, we have made a number of interesting observations. We have found that short deletions in microsatellite regions, especially 1 bp deletions, are consistently highly over-represented in MSI cancer cell lines compared to MSS cancer cell lines and immortalized but non-malignant HapMap samples. On the other hand, we did not find any consistent increase in longer deletions or insertions in MSI samples. A similar observation has been made in yeast [9], however to the best of our knowledge it is the first time this phenomenon is reported in human cells. This observation confirms that multiple DNA repair mechanisms are at play in normal cells and that within microsatellites, accidental deletions and insertions of different lengths are repaired through distinct mechanisms.

Another observation is that that there are more microsatellite indels in coding regions in cancer cells and especially in MSI-positive cancer cells. This discovery is surprising since a substantial fraction of these indels are expected to cause internal frameshifts, non-sense mediated decay and therefore gene inactivation. Our results on the other hand suggest that cancer cells with increased coding microsatellite indels might be positively selected and therefore that coding microsatellite indels could contribute to the tumor phenotypes. Alternatively, these indels might generate functional diversity that allows rapid adaptation of the tumor cells to changing environments, perhaps similar to what has been observed in yeast [20].

Our method is based on transcriptome sequencing, which has some limitations when used for MSI characterization. For instance, it will miss altered microsatellites located in regulatory sequences, which may also play important roles in oncogenesis. Moreover, as indel detection requires adequate read coverage, it may be difficult to detect microsatellite indels in transcripts with low abundances. Despite above disadvantages, inspection of expressed transcripts still provides significant information about MMRD. As RNA-seq experiments are widely performed and the price for sequencing cancer samples is rapidly decreasing, in the future RNA-seq based diagnosis method will become cost effective for clinical applications. Moreover, one single RNA-Seq assay is able to provide accurate MSI diagnosis along with rich information about the many other aspects of tumor, including functional mutations, gene expression profiles, active pathways and gene fusions, etc, making specialized PCR assays for MSI unnecessary.

In conclusion, our method is the first to enable genome-wide characterization of MMRD status in human cell through the integration of high-throughput sequencing data. Consistent with previous findings, we have observed a significant increase of short deletions in MSI cells compared to MSS ones. Based on this observation, we showed that the PI/PD ratio (which we also define as MSI-seq) can be used to quantify MSI status in both cancer cell lines and primal tumor samples from patients, and has multiple advantages over traditional assays. Therefore, our method has the potential of serving as a novel diagnosis tool of genomic instability for different cancer samples.

## References

[1] Sinicrope FA, Sargent DJ (2012) Molecular pathways: microsatellite instability in colorectal cancer: prognostic, predictive, and therapeutic implications. Clinical cancer research 18: 1506–1512 doi:10.1158/1078-0432.CCR-11-1469. 22302899 10.1158/1078-0432.CCR-11-1469 PMC3306518

[2] De la Chapelle A (2003) Microsatellite instability. The New England journal of medicine 349: 209–210 doi:10.1056/NEJMp038099. 12867603 10.1056/NEJMp038099

[3] Chen Y, Wang J, Fraig MM, Metcalf J, Turner WR, et al (2001) Defects of DNA mismatch repair in human prostate cancer. Cancer research 61: 4112–4121. 11358834

[4] Wacker SA, Houghtaling BR, Elemento O, Kapoor TM (2012) Using transcriptome sequencing to identify mechanisms of drug action and resistance. Nature chemical biology 8: 235–237 doi:10.1038/nchembio.779. 22327403 10.1038/nchembio.779 PMC3281560

[5] Dietmaier W, Wallinger S, Bocker T (1997) Diagnostic Microsatellite Instability: Definition and Correlation with Mismatch Repair Protein Expression. Cancer Research: 4749–4756. 9354436

[6] Boland CR, Thibodeau SN, Hamilton SR, Sidransky D, Eshleman JR, et al (1998) A National Cancer Institute Workshop on Microsatellite Instability for cancer detection and familial predisposition: development of international criteria for the determination of microsatellite instability in colorectal cancer. Cancer research 58: 5248–5257. 9823339

[7] Sun X, Chen C, Vessella RL, Dong J (2006) Microsatellite instability and mismatch repair target gene mutations in cell lines and xenografts of prostate cancer. The Prostate 66: 660–666 doi:10.1002/pros.20390. 16388502 10.1002/pros.20390

[8] Faulkner RD, Seedhouse CH, Das-Gupta EP, Russell NH (2004) BAT-25 and BAT-26, two mononucleotide microsatellites, are not sensitive markers of microsatellite instability in acute myeloid leukaemia. British journal of haematology 124: 160–165. 14687025 10.1046/j.1365-2141.2003.04750.x

[9] Sia EA, Kokoska RJ, Dominska M, Greenwell P, Petes TD (1997) Microsatellite instability in yeast: dependence on repeat unit size and DNA mismatch repair genes. Molecular and cellular biology 17: 2851–2858. 9111357 10.1128/mcb.17.5.2851 PMC232137

[10] Pickrell JK, Marioni JC, Pai AA, Degner JF, Engelhardt BE, et al (2010) Understanding mechanisms underlying human gene expression variation with RNA sequencing. Nature 464: 768–772 doi:10.1038/nature08872. 20220758 10.1038/nature08872 PMC3089435

[11] Montgomery SB, Sammeth M, Gutierrez-Arcelus M, Lach RP, Ingle C, et al (2010) Transcriptome genetics using second generation sequencing in a Caucasian population. Nature 464: 773–777 doi:10.1038/nature08903. 20220756 10.1038/nature08903 PMC3836232

[12] Seshagiri S, Stawiski EW, Durinck S, Modrusan Z, Storm EE, et al (2012) Recurrent R-spondin fusions in colon cancer. Nature 488: 660–664 doi:10.1038/nature11282. 22895193 10.1038/nature11282 PMC3690621

[13] Benson G (1999) Tandem repeats finder: a program to analyze DNA sequences. Nucleic acids research 27: 573–580. 9862982 10.1093/nar/27.2.573 PMC148217

[14] Li H, Durbin R (2010) Fast and accurate long-read alignment with Burrows-Wheeler transform. Bioinformatics 26: 589–595 doi:10.1093/bioinformatics/btp698. 20080505 10.1093/bioinformatics/btp698 PMC2828108

[15] Albers CA, Lunter G, Macarthur DG, McVean G, Ouwehand WH, et al (2011) Dindel: Accurate indel calls from short-read data. Genome research 21: 961–973 doi:10.1101/gr.112326.110. 20980555 10.1101/gr.112326.110 PMC3106329

[16] Sherry ST, Ward MH, Kholodov M, Baker J, Phan L, et al (2001) dbSNP: the NCBI database of genetic variation. Nucleic acids research 29: 308–311. 11125122 10.1093/nar/29.1.308 PMC29783

[17] Trapnell C, Williams BA, Pertea G, Mortazavi A, Kwan G, et al (2010) Transcript assembly and quantification by RNA-Seq reveals unannotated transcripts and isoform switching during cell differentiation. Nature biotechnology 28: 511–515 doi:10.1038/nbt.1621. 10.1038/nbt.1621 PMC3146043 20436464

[18] Rajadhyaksha AM, Elemento O, Puffenberger EG, Schierberl KC, Xiang JZ, et al (2010) Mutations in FLVCR1 cause posterior column ataxia and retinitis pigmentosa. American journal of human genetics 87: 643–654 doi:10.1016/j.ajhg.2010.10.013. 21070897 10.1016/j.ajhg.2010.10.013 PMC2978959

[19] Jiang Y, Soong TD, Wang L, Melnick AM, Elemento O (2012) Genome-wide detection of genes targeted by non-Ig somatic hypermutation in lymphoma. PloS one 7: e40332 doi:10.1371/journal.pone.0040332. 22808135 10.1371/journal.pone.0040332 PMC3395700

[20] Verstrepen KJ, Jansen A, Lewitter F, Fink GR (2005) Intragenic tandem repeats generate functional variability. Nature genetics 37: 986–990 doi:10.1038/ng1618. 16086015 10.1038/ng1618 PMC1462868

[21] Seitz S, Wassmuth P, Plaschke J, Schackert HK, Karsten U, et al (2003) Identification of microsatellite instability and mismatch repair gene mutations in breast cancer cell lines. Genes, chromosomes & cancer 37: 29–35 doi:10.1002/gcc.10196. 12661003 10.1002/gcc.10196

[22] Laghi L, Bianchi P, Delconte G, Celesti G, Di Caro G, et al (2012) MSH3 protein expression and nodal status in MLH1-deficient colorectal cancers. Clinical cancer research 18: 3142–3153 doi:10.1158/1078-0432.CCR-12-0175. 22496206 10.1158/1078-0432.CCR-12-0175

[23] Yeh CC, Lee C, Dahiya R (2001) DNA mismatch repair enzyme activity and gene expression in prostate cancer. Biochemical and biophysical research communications 285: 409–413 doi:10.1006/bbrc.2001.5187. 11444857 10.1006/bbrc.2001.5187

[24] Boyer JC, Umar A, Risinger JI, Lipford JR, Kane M, et al (1995) Microsatellite instability, mismatch repair deficiency, and genetic defects in human cancer cell lines. Cancer research 55: 6063–6070. 8521394

[25] Van der Heijden MS, Brody JR, Elghalbzouri-Maghrani E, Zdzienicka MZ, Kern SE (2006) Does tumorigenesis select for or against mutations of the DNA repair-associated genes BRCA2 and MRE11?: considerations from somatic mutations in microsatellite unstable (MSI) gastrointestinal cancers. BMC genetics 7: 3 doi:10.1186/1471-2156-7-3. 16417627 10.1186/1471-2156-7-3 PMC1382246

[26] Griffith M, Griffith OL, Mwenifumbo J, Goya R, Morrissy AS, et al (2010) Alternative expression analysis by RNA sequencing. Nature methods 7: 843–847 doi:10.1038/nmeth.1503. 20835245 10.1038/nmeth.1503

[27] Sam LT, Lipson D, Raz T, Cao X, Thompson J, et al (2011) A comparison of single molecule and amplification based sequencing of cancer transcriptomes. PloS one 6: e17305 doi:10.1371/journal.pone.0017305. 21390249 10.1371/journal.pone.0017305 PMC3046973

[28] Prensner JR, Iyer MK, Balbin OA, Dhanasekaran SM, Cao Q, et al (2011) Transcriptome sequencing across a prostate cancer cohort identifies PCAT-1, an unannotated lincRNA implicated in disease progression. Nature biotechnology 29: 742–749 doi:10.1038/nbt.1914. 10.1038/nbt.1914 PMC3152676 21804560

[29] Edgren H, Murumagi A, Kangaspeska S, Nicorici D, Hongisto V, et al (2011) Identification of fusion genes in breast cancer by paired-end RNA-sequencing. Genome biology 12: R6 doi:10.1186/gb-2011-12-1-r6. 21247443 10.1186/gb-2011-12-1-r6 PMC3091304

[30] Wang ET, Sandberg R, Luo S, Khrebtukova I, Zhang L, et al (2008) Alternative isoform regulation in human tissue transcriptomes. Nature 456: 470–476 doi:10.1038/nature07509. 18978772 10.1038/nature07509 PMC2593745

[31] Berger MF, Levin JZ, Vijayendran K, Sivachenko A, Adiconis X, et al (2010) Integrative analysis of the melanoma transcriptome. Genome research 20: 413–427 doi:10.1101/gr.103697.109. 20179022 10.1101/gr.103697.109 PMC2847744

[32] Matheson EC, Hall AG (2003) Assessment of mismatch repair function in leukaemic cell lines and blasts from children with acute lymphoblastic leukaemia. Carcinogenesis 24: 31–38. 12538346 10.1093/carcin/24.1.31

[33] Koeppel M, Van Heeringen SJ, Kramer D, Smeenk L, Janssen-Megens E, et al (2011) Crosstalk between c-Jun and TAp73{alpha}/{beta} contributes to the apoptosis-survival balance. Nucleic acids research 39: 6069–6085 doi:10.1093/nar/gkr028. 21459846 10.1093/nar/gkr028 PMC3152320

[34] Lee S, Seo CH, Lim B, Yang JO, Oh J, et al (2011) Accurate quantification of transcriptome from RNA-Seq data by effective length normalization. Nucleic acids research 39: e9 doi:10.1093/nar/gkq1015. 21059678 10.1093/nar/gkq1015 PMC3025570

